# TRAF6 regulates EGF-induced cell transformation and cSCC malignant phenotype through CD147/EGFR

**DOI:** 10.1038/s41389-018-0030-1

**Published:** 2018-02-20

**Authors:** Xu Zhang, Lisha Wu, Ta Xiao, Ling Tang, Xuekun Jia, Yeye Guo, JiangLin Zhang, Jie Li, Yijing He, Juan Su, Shuang Zhao, Juan Tao, Jianda Zhou, Xiang Chen, Cong Peng

**Affiliations:** 1 The Department of Dermatology, Xiangya Hospital, Central South University, Changsha, Hunan China; 2 Hunan Key Laboratory of Skin Cancer and Psoriasis, Xiangya Hospital, Central South University, Changsha, Hunan China; 3 Department of Dermatology, Affiliated Union Hospital, Tongji Medical College, Huazhong University of Science and Technology, Wuhan, China; 4 Department of Plastic Surgery of Third Xiangya Hospital, Central South University, Changsha, China

## Abstract

TRAF6, a well-known adapter molecule, plays pivotal role in TLR/IL-1R associated signaling pathway. Although TRAF6 has been shown to have oncogenic activity in various malignant tumors, the details remain unclear. In this study, we demonstrated that TRAF6 facilitates Ras (G12V) and EGF-induced cellular transformation through EGFR. Silencing of TRAF6 expression significantly downregulated AP-1 activity, as well as MMP-2,9 expression after EGF stimulation. Furthermore, we found that TRAF6 plays an essential role in cutaneous squamous cell carcinoma (cSCC) malignant phenotypes, affecting cell growth and migration. CD147/Basigin, a transmembrane glycoprotein belonging to the immunoglobulin superfamily, is over-expressed in tumors and induces tumorigenesis. Our results showed that CD147 formed complex with EGFR and TRAF6. Knockdown of TRAF6 disrupted the CD147-EGFR complex, thereby inducing EGFR endocytosis. Therefore, TRAF6 might be a novel molecular target for cSCC prevention or therapy.

## Introduction

Cutaneous squamous cell carcinoma (cSCC) is second incidence to basal cell carcinoma (BCC) among malignant non-melanoma skin cancers^1^. Although the incidence of cSCC is much lower than that of BCC in the general population, the clinical characteristics of cSCC manifest more aggressively. cSCC refers to the cellular malignant transformation and abnormal growth of keratinocyte cells, which are the major cell type in the epidermis. Unlike BCC, cSCC also shows aggressive behavior, which showed the risk for metastasis relevent to advanced high-risk lesions with, and ~4.0–12.5% of patients have nodal metastasis^2, 3^. In addition, the increased incidence of recurrence and metastasis after surgical resection is associated with several features, including tumor diameters of >2 cm, deep invasion (>2 mm), localization to chronically damaged or diseased skin and poor histological differentiation^4^.

Most cSCC occurs on the head, neck, and extremities, where there is a large possibility of exposure to the sun, as ultraviolet (UV) exposure is the major cause and a direct contributor to the occurrence of cSCC. cSCC pathogenesis follows the classic tumor model, involving multiple steps from precancerous lesion, such as actinic keratosis (AK) to carcinoma in situ, final to invasive of cSCC. Subsequent steps include malignant transformation, abnormal cell growth, angiogenesis, invasion of the surrounding tissue and formation distant organ metastasis.

EGFR (epidermal growth factor receptor) is a transmembrane member of the ErbB receptor tyrosine kinase family, which is located on the cellular surface^5^. After associating with its ligands, such as EGF or transforming growth factor-a (TGF-a), EGFR dimerizes and triggers auto-phosphorylation of tyrosine kinases, thus leading to activation of various intracellular downstream pathways, including the PI3K/AKT, RAF/MEK/ERK, and STAT3 signaling pathways^6^. Dysregulation of EGFR is closely linked to tumorigenesis and has been implicated in a number of tumors^7–11^. EGFR is over-expressed on cSCC cells, particularly in advanced or metastatic tumor tissue^12–14^. Genetic analysis has indicated that EGFR has a very low frequency of mutation in cSCC^15, 16^, and RAS mutations are also very rarely observed in cSCC^16, 17^. Mutated RAS may activate molecules downstream of EGFR, and consequently, inhibition of EGFR is largely ineffective in tumor entities with RAS mutations. Because of dysregulated EGFR activation in the absence of EGFR or RAS mutations, targeting EGFR is a promising therapeutic strategy in cSCC^18–20^.

Tumor necrosis factor receptor-associated factor 6 (TRAF6), a member of the TRAF family, was first identified as an adaptor of the signals induced by the TNFR. The TRAF family comprises signal transducers of TLR/interleukin-1 (IL-1) family members, which triggers signaling transduction in innate immune responses^21^. In addition, TRAF6 has an E3 ubiquitin ligase activity mediatesd conjugation of lysine-63 (K63)–linked polyubiquitin chains to proteins^22, 23^. Recent studies have reported that TRAF6 promotes oncogenesis by inhibiting apoptosis and stimulating proliferation and invasion in cancer. TRAF6 alters the expression of Bcl2, Bax, and MMP9, thereby regulating cell apoptosis and invasive ability in gastric cancer^24^. TRAF6 upregulates HIF-1a expression and promote tumor angiogenesis in colon cancer^25^. Luo et al. have demonstrated that TRAF6 directly interacts with CD147, thereby promoting melanoma invasion and metastasis, whereas inhibition of TRAF6 expression or activity reverses the malignant phenotype of melanoma cells^26, 27^. In addition, TRAF6 is highly expressed in human pancreatic cancer^28^, colon cancer^29^, gliomas^30^, breast cancer^31^, and lung cancer^32^. However, the role of TRAF6 in cSCC remains unknown. In this study, we found that TRAF6 is required for EGF-induced cell transformation and plays critical roles in cSCC cell growth and metastasis through EGFR signaling pathways.

## Results

### TRAF6 mediates EGF-induced cell transformation and cell migration

**Fig. 1:**
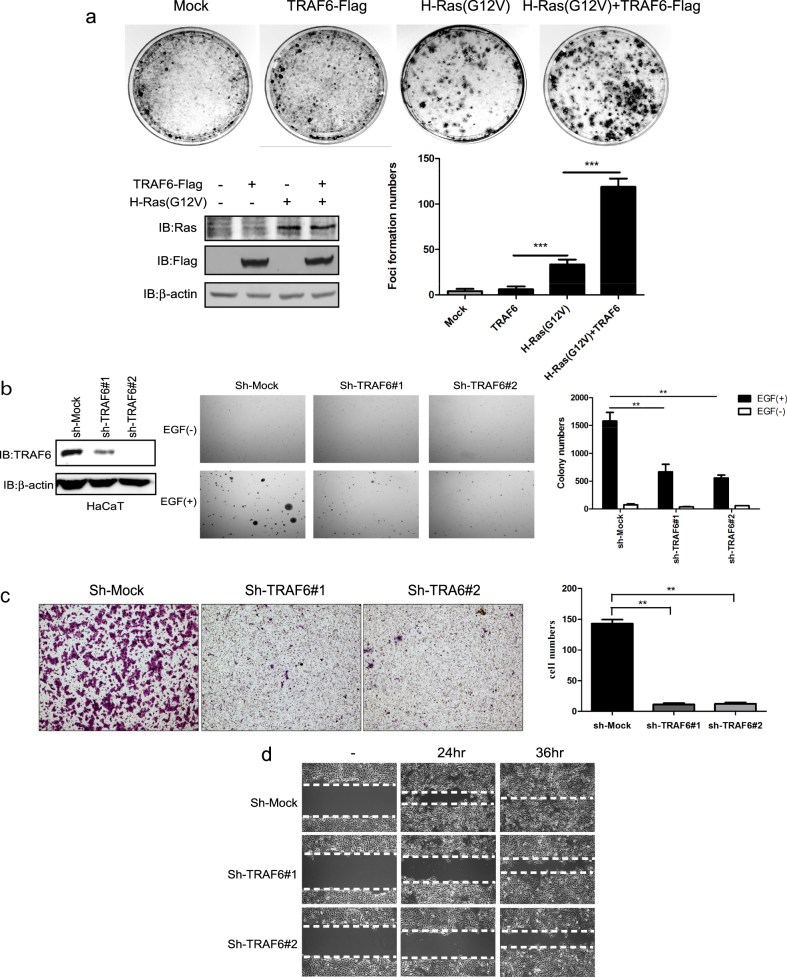
TRAF6 mediates EGF-induced anchorage-independent neoplastic cell transformation and cell migration. **a** Ectopic TRAF6 promotes Ras (G12V)-induced foci formation. TRAF6, Ras (G12V) and TRAF6 together with Ras (G12V) were transfected into NIH3T3 cells as indicated, and a foci formation assay was performed as described in Materials and methods (upper panel). The histograms showed the result of three times repeats are calculated as means ± S.D. Significant differences were evaluated using a one-way ANOVA and the asterisk (*) indicates a significant difference (*p* < 0.05) (lower panel). The protein levels of transfected TRAF6 and Ras (G12V) were tested by western blotting with the indicated antibodies (lower panel). **b** Knockdown of TRAF6 attenuates EGF-induced cell transformation. Stable TRAF6-silenced HaCaT cells were generated as described in Materials and methods. Cells were seeded in 0.3% BME agar containing 20% FBS plus EGF (20 ng/ml) and maintained in a 37 °C, 5% CO_2_ incubator for 12 d, and colonies were counted using a microscope and the ImageJ computer software program. Representative photos are shown, and data from multiple experiments are expressed as the means ± S.D. The asterisk (*) indicates a significant difference between cells expressing *mock* or *sh-TRAF6* (*p* < 0.05, Student’s *t-*test). **c**, **d** The effect of TRAF6 on cell migration. Cells that migrated across the membrane were stained with crystal violet and imaged at ×100 magnification. The multiple areas (*n* = 5) represent the means ± S.D. of each group. The asterisk (*) indicates a significant difference between cells expressing *mock* or *sh-TRAF6* (*p* < 0.05, Student’s *t-*test), as shown in **c**. Wound healing assays were conducted to test the migration capability in vitro as described in Materials and methods. Images (at ×40 magnification) were taken at 24 h and 36 h, as shown in **d**

TRAF6 has a critical function in the LPS/IL-1β-induced signaling pathway through the TAK1-Ikkα/β pathway, but whether TRAF6 is involved in the oncogenic stimuli-induced transduction pathway remains unknown. Our findings showed that TRAF6 promotes Ras (G12V)-induced cell transformation in NIH3T3 cells. The NIH3T3 cells had been transfected with TRAF6 alone, Ras (G12V) alone or TRAF6 with Ras (G12V). As shown in Fig. 1a, overexpression of Ras (G12V) alone could induce foci formation clearly, whereas TRAF6 failed to induce NIH3T3 cells foci formation. However, the combination of TRAF6 with Ras (G12V) induced more foci formation than Ras (G12V) alone (Fig. 1a), thus indicating that TRAF6 may play a role in cell transformation. To clarify the role of TRAF6 in cell transformation, we constructed stable TRAF6-silenced HaCaT cells by using two independent targeting sequences (Fig. 1b, left panel). As expected, EGF mediated anchorage-independent cell growth was significantly blocked in TRAF6 expresison inhibition HaCaT cells (Fig. 1b, right panel). In addition, wound healing and Transwell assays were performed to study the function of TRAF6 in cell migration. The number of migratory cells was dramatically decreased in TRAF6-knockdown HaCaT cells (Fig. 1c, d). In addition, overexpression of TRAF6-wt could promote cell proliferation and migration, but not for TRAF6-DN in HaCaT cells (sFig. 1), which suggest that the activity of TRAF6 regulates cell transformation.

### TRAF6 has critical roles in cSCC malignant phenotype

**Fig. 2:**
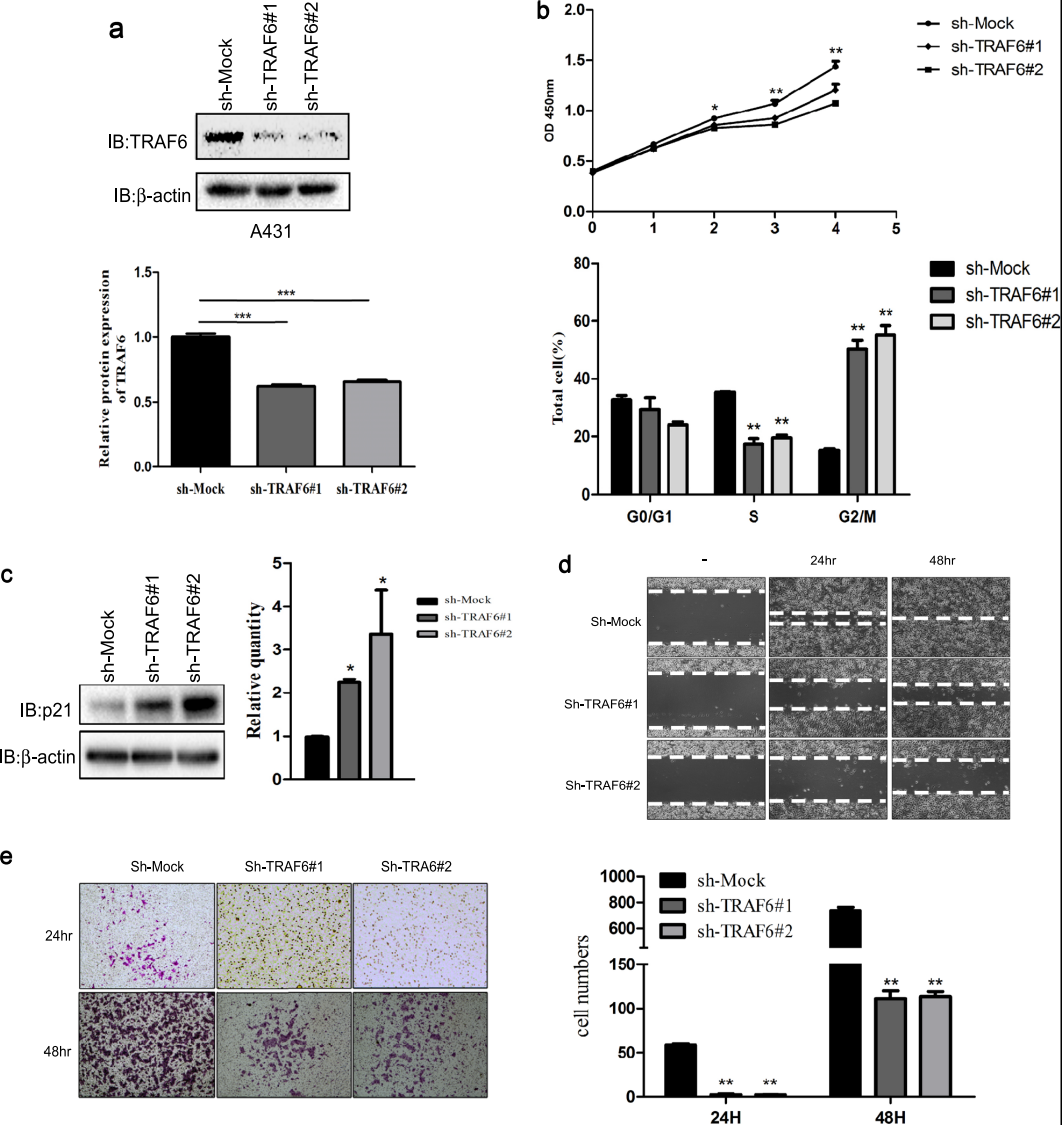
TRAF6 affects the cSCC malignant phenotype. **a** Knockdown of TRAF6 attenuates the proliferation of A431 cells. TRAF6-knockdown cells were generated by using different sequences as described in Materials and methods. TRAF6 expression was assessed by western blotting as indicated (upper panel). Histograms indicated relative TRAF6 expression, as means ± S.D. Significant differences were evaluated using a one-way ANOVA and the asterisk (*) indicates a significant difference (*p* < 0.05) (lower panel). **b** The TRAF6-silenced cells were seeded into 96-well plates, and proliferation was assessed by using a CellTiter96 Aqueous One Solution detection kit. The data from multiple experiments are expressed as the means ± S.D. Significant differences were evaluated using one-way ANOVA, and the asterisk (*) indicates a significant difference (*p* < 0.05) (upper panel). The effect of TRAF6 knockdown on cell cycle distribution. *sh-Mock, sh-TRAF6#1*&*#2* cells were analyzed by FCM as described in Materials and methods (lower panel). **c** The protein level and transcriptional expression of p21 were measured by western-bloting and RT-real time PCR as described in Materials and methods. The data from triplicate experiments are expressed as the means ± S.D. Significant differences were evaluated with one-way ANOVA, and significant differences are indicated, as shown in (**c**). **d**, **e** Wound healing assays were conducted to test the migration capability in vitro as described in Materials and methods. Images (at ×40 magnification) were taken at 24 h and 48 h, as shown in (**d**). The effect of TRAF6 on cSCC cell migration. Cells that migrated across the membrane were stained with crystal violet and imaged at ×100 magnification. Data represent the means (*n* = 3) ± S.D. of each group. The asterisk (*) indicates a significant difference between cells expressing *mock* or *sh-TRAF6* (*p* < 0.05, Student’s *t*-test), as shown in (**e**)

Cell transformation is a critical step in the process of cSCC, and our above data showed that TRAF6 affects Ras (G12V) and EGF-induced cell transformation, as well as cell migration in HaCaT cells, thus suggesting that TRAF6 might be involved in cSCC pathogenesis. To investigate the effect of TRAF6 on the cSCC malignant phenotype, we generated a stable TRAF6-knockdown A431 cell line (Fig. 2a). As shown in the right panel of Fig. 2b (upper panel), cell proliferation was significantly suppressed in TRAF6-silenced cells. Furthermore, knockdown of TRAF6-induced cell cycle G2/M arrest (Fig. 2b, lower panel) and raised p21 protein level (Fig. 2c, left panel), as well as transcriptional expression (Fig. 2c, right panel). As mentioned previously, ~4–10% of cSCC patients had distant metastasis, and therefore, we also tested the effect of TRAF6 on tumor cell migration. In agreement with our previous results, the migration of A431 cells was dramatically decreased after TRAF6 knockdown (Fig. 2d, e).

**Fig. 3:**
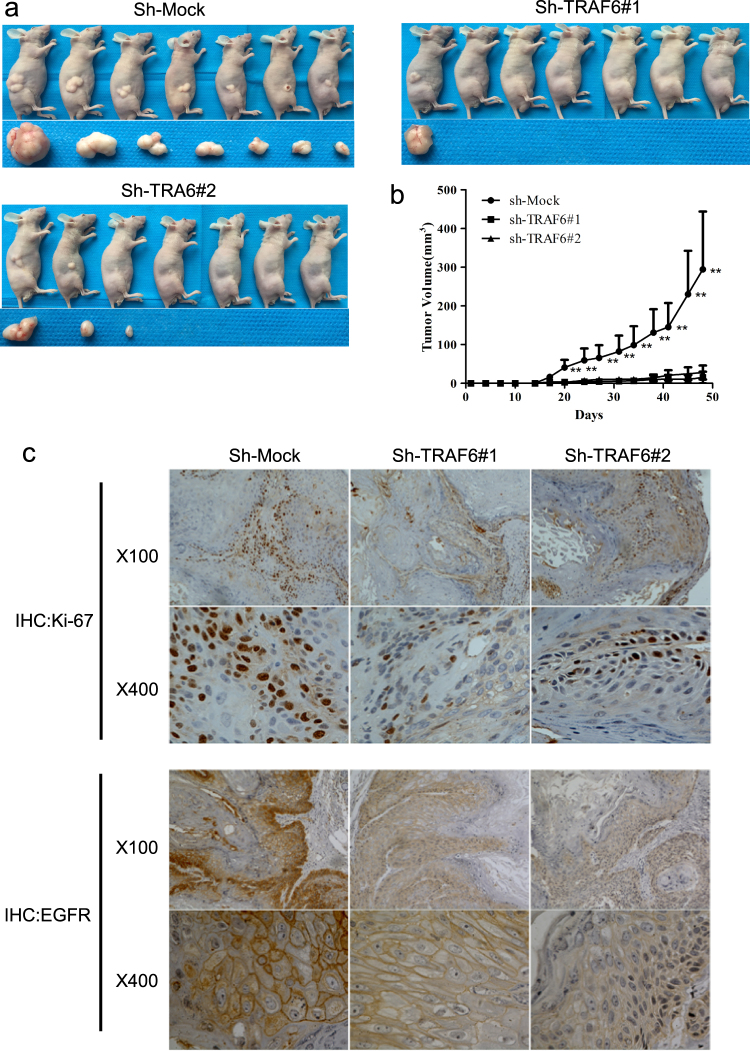
Knockdown of TRAF6 blocks the growth of A431 cell-derived tumors in a xenograft mouse model. **a**, **b** sh-Mock or sh-TRAF6 #1,2 cells (1 × 10^6^) were injected into the right flanks of female athymic nude mice. The growth of tumors was monitored as described in Materials and methods. The results are shown as mean tumor volume ± S.D., and the asterisk (*) indicates a significant difference (*p* < 0.05 one-way ANOVA). **c** Tumor tissues were isolated from nude mice and immunohistochemistry (IHC) was conducted as described in Materials and methods to assess Ki-67 and EGFR expression

To validate the effect of TRAF6 on cSCC growth, we constructed xenograft models to test the role of TRAF6 in cell proliferation in vivo. In agreement with the in vitro data, silencing TRAF6 expression significantly blocked cSCC cell growth, thus resulting in smaller tumor sizes and delayed tumor formation (Fig. 3a, b). Histopathological findings of the tumors showed a decreased Ki-67 and EGFR expression in the knockdown of TRAF6 group, compared with control group (Fig. 3c). These results suggested that decreased levels of TRAF6 may lead to comparable attenuation of tumor growth via decreased cell proliferation.

### TRAF6 regulates EGF-induced signaling pathways through EGFR

**Fig. 4:**
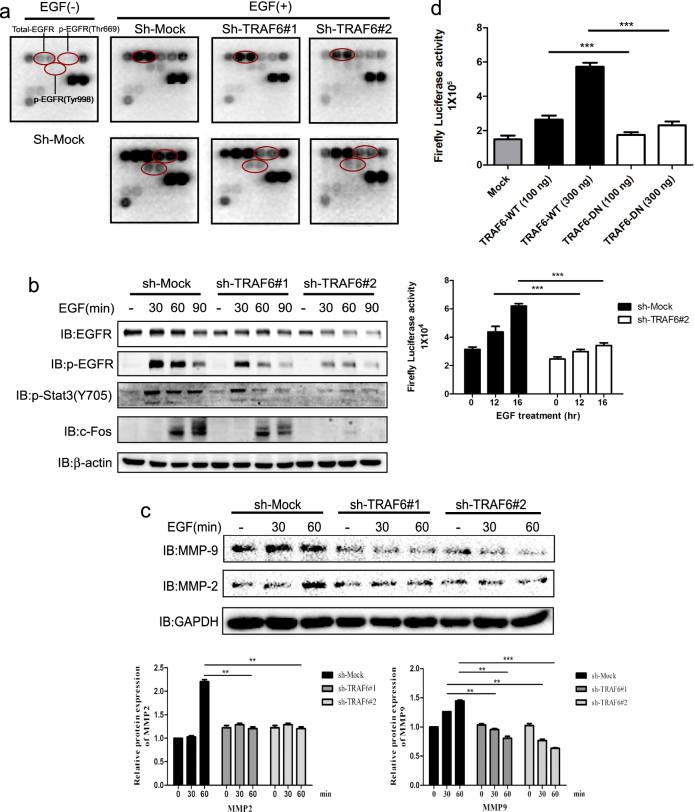
TRAF6 regulates the EGF-induced signaling pathway through EGFR. **a**, **b**, **c** Silencing of TRAF6 attenuates p-EGFR and EGFR expression. HaCaT cells expressing *sh-TRAF6#1,2* were starved for 36 h and then treated with EGF (100 ng/ml) for 30 min, and a protein array was performed to assess the effect of TRAF6 on the EGFR signaling pathway (**a**) or cells expressing sh-TRAF6 were starved for 36 h and then treated with EGF (100 ng/ml) for various times, as indicated. Immunoblotting was used to detect protein expression with the indicated antibodies. Anti-β-Actin or Gad were used to verify equal loading of protein (**b**, **c**). The histograms indicated relative MMP-2 and MMP-9 expression, as means ± S.D. Significant differences were evaluated using a one-way ANOVA and the asterisk (*) indicates a significant difference (*p* < 0.05) (lower panel). **d** TRAF6 regulates AP-1 activity. Flag-TRAF6-WT and Flag-TRAF6-DN cells were transfected with the *AP-1-luciferase reporter* gene (100 ng), as well as the *Renilla luciferase* gene (20 ng) for normalization. At 30 h after transfection, the firefly luciferase activity was determined in cell lysates and normalized to the *Renilla luciferase* activity. Significant differences were evaluated using Student’s *t*-test, and the respective asterisks indicate a significant difference (*p* < 0.05) (upper panel). TRAF6-silenced cells were co-transfected with a plasmid mixture containing the *AP-1 luciferase reporter* gene (0.8 µg) and the *Renilla luciferase* gene (0.2 µg) for normalization. At 20 h after transfection, cells were starved for 16 h and then treated with EGF (20 ng/mL) for various times, as indicated. Firefly luciferase activity was determined in cell lysates and normalized against *Renilla luciferase* activity. Significant differences were evaluated using Student’s *t*-test, and the respective asterisks indicate a significant difference (*p* < 0.05) (lower panel)

Our previous results indicated that TRAF6 promotes Ras (G12V) and EGF-induced cell transformation, as well as a malignant phenotype in A431 cells, thus suggesting that TRAF6 might, to some extent, exert role in EGF pathway. To investigate the details of the role of TRAF6 in the EGFR pathway, we used an EGFR Signaling Antibody Array and found that the expression of total EGFR and p-EGFR was significantly decreased in TRAF6-knockdown HaCaT cells (Fig. 4a). To confirm the effect of TRAF6 on EGF-induced pathways, we assessed the activation of key molecules in TRAF6-knockdown HaCaT cells EGF treatment. The results showed that silence TRAF6-expression attenuated the phosphorylation of EGFR, Stat3 and blocked c-Fos expression in presence of EGF (Fig. 4b). EGFR is known to trans-activate MMPs, such as MMP-2,9^33–35^. Given TRAF6 regulation of EGFR, we next analyzed MMP-2,9 expression in TRAF6-silenced HaCaT cells after EGF treatment. As shown in Fig. 4c, knockdown of TRAF6 suppressed EGF-induced MMP-2,9 expression.

The dimeric AP-1 complex, which may include Jun, Fos, and other family members, is a critical downstream mediator of the EGF-induced transduction pathway. To explore whether TRAF6 might affect AP-1 activity, HEK293 cells were transiently transfected with the *AP-1* reporter gene with different amount of *TRAF6-WT* or *TRAF6-DN* (ring domain mutation), as shown in Fig. 4d (upper panel), the activity of *AP-1* had been dramatically raised with dose-dependent manner in cells expressing TRAF6-WT but not in those expressing TRAF6-DN. Next, to confirm the effect of TRAF6 on *AP-1* activity, the *AP-1* reporter gene together with *Renilla luciferase* gene were transfected into TRAF6 expression silence HaCaT cells and found that *AP-1* activity was downregulated in TRAF6 low-expression HaCaT cells (Fig. 4d, lower panel).

### TRAF6 regulates EGFR activation through CD147

**Fig. 5:**
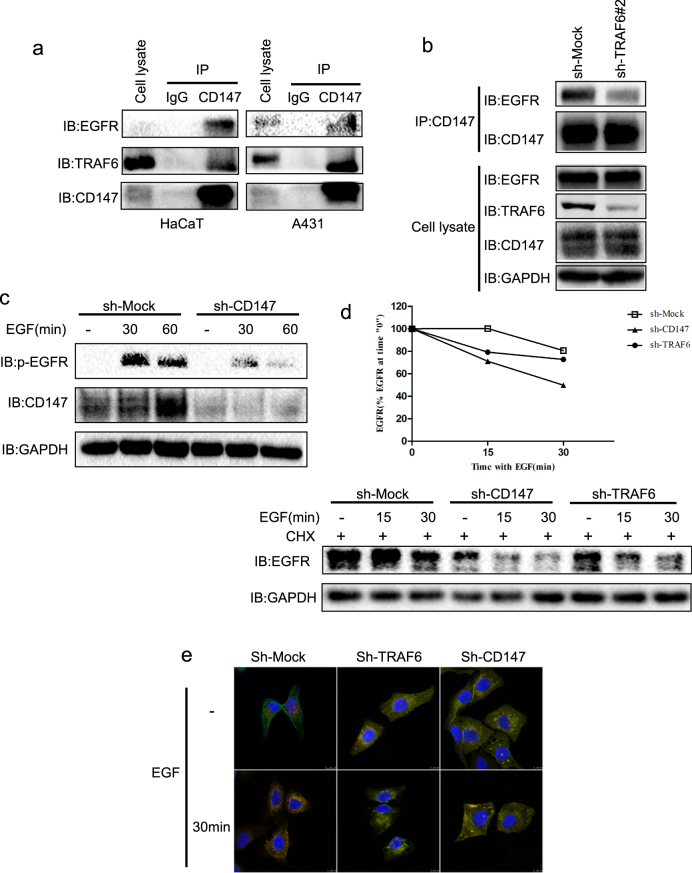
TRAF6 regulates EGFR expression through CD147. **a** CD147 interacts with TRAF6 and EGFR. HaCaT cell and A431 cell extracts were used for immunoprecipitation with a CD147 antibody or control IgG. The immunoprecipitated complex was detected by western blotting with the indicated antibodies. **b** TRAF6 affects CD147 binding to EGFR. The extracts from TRAF6-silenced cells were immunoprecipitated with anti-CD147 antibody or control IgG, and then, WB was performed to detect EGFR expression. **c** CD147 regulates EGFR in the EGF-induced signaling pathway. HaCaT cells expressing *sh-CD147* were starved for 36 h and then treated with EGF (100 ng/ml) for various time points. Immunoblotting was used to detect EGFR expression, and anti-Gad was used to verify equal loading of protein. **d** TRAF6 and CD147 regulate EGFR-protein stability. Cells expressing *Sh-TRAF6* and *sh-CD147* were constructed as described in “Materials and methods”. After serum starvation for 16 h, the cells were treated with CHX and then stimulated with EGF (100 ng/ml) for various time points, as indicated. Immunoblotting was used to detect EGFR expression, and anti-GAPDH was used to verify equal loading of protein. **e** TRAF6 and CD147 alter EGFR endocytosis. Cells expressing *Sh-TRAF6* and *sh-CD147* were seeded onto cover glasses. After serum starvation for 16 h, the cells were stimulated with EGF (100 ng/ml) for various time points, as indicated. The cells were then fixed and stained using an anti-EGFR fluorescence-conjugated antibody. The cover glasses were mounted and visualized by using confocal microscopy. The green color indicates endogenous EGFR staining in cells. Three independent experiments were performed and showed similar results

To study the mechanism associated with TRAF6 regulation of EGFR, we tested the direct interaction between TRAF6 and EGFR, but the experiment failed (data not shown). Our previous results had demonstrated that TRAF6 directly interacts with CD147. CD147 is a cell surface protein belonging to the immunoglobulin super-family involved various tumor malignant phenotypes^36–38^. Silencing CD147 expression with siRNA or anti-CD147 antibody has been shown to significantly attenuate total EGFR expression or phosphorylation of EGFR^39–41^, thus indicating that CD147 is a key EGFR mediator. Therefore, we propose that CD147 acts as a “bridge” connecting TRAF6 and EGFR. To test this hypothesis, we performed endogenous immunoprecipitation with CD147 and found that TRAF6 and EGFR were detected in the CD147 complex (Fig. 5a), thus indicating that CD147 interacts with TRAF6 and EGFR. Interestingly, knockdown of TRAF6 impaired CD147 binding to EGFR (Fig. 5b). We also examined the effect of CD147 on phosphorylation of EGFR, and in agreement with the previous results, knockdown of CD147 dramatically blocked p-EGFR with EGF treatment (Fig. 5c).

Under ligand stimulation, endocytosis of EGFR occurs and leads to a shift from diffuse membrane staining to punctate cytoplasmic staining (representing endocytic vesicles). To understand the role of TRAF6 and CD147 in EGFR trafficking, we utilized a fluorescence-conjugated primary antibody to track EGFR and examined the effect of TRAF6, CD147 on EGFR protein stability. We found that knockdown of TRAF6 and CD147 accelerated EGFR-protein degradation (Fig. 5d) and EGFR-containing endocytic vesicles were present in TRAF6- and CD147-knockdown HaCaT cells even without EGF treatment (Fig. 5e), because the EGFR distribution typically shifts from the cell membrane to the cytoplasm, with punctate fluorescence after EGF stimulation in control cells (Fig. 5e). Hence, our results suggested that TRAF6 and CD147 may be involved in EGFR endocytosis.

## Discussion

Epidermal cell transformation plays a critical stage in cSCC development that involves oncogenic key molecules. In this study, we found that TRAF6 plays a key role in cell transformation. Although over-expression of TRAF6 alone did not effect on foci formation in NIH3T3 cells, more foci were observed with a combination of TRAF6 and Ras (G12V), thus indicating that TRAF6 enhances the cell transformation ability driven by oncogenes (Fig. 1a). SliencingTRAF6 expresison significantly suppressed the EGF meidated anchorage-independent cell growth of HaCaT cells (Fig. 1b), thus indicating that TRAF6 is involved in the EGF associated signaling pathway. Regarding the TLR/IL-1β pathway, TRAF6 is also an essential molecule for activation of other signaling pathways. After TGFβ stimulation, TRAF6 induces Lys63-linked ubiquitination of TβR1, which in turn promotes cleavage of TβR1 by TACE. After cleavage, the intracellular domain (ICD) is released from TβR1, translocates into the nucleus and subsequently forms a complex with p300 that regulates expression of genes, such as Snail, MMP2, and cyclinD1^42–44^. In the IGF-induced pathway, TRAF6 acts as a direct E3 ligase, ubiquitinating AKT, and thereby inducing AKT cellular membrane recruitment and phosphorylation leading to activation^45^.

In this study, we provided the first evidence that TRAF6 affects the EGF-induced pathway. Through a protein array, we found that p-EGFR and total EGFR were altered in TRAF6-silenced cells (Fig. 4a). Furthermore, p-Stat3, p-EGFR, and c-Fos were dramatically downregulated by TRAF6 knockdown (Fig. 4b). EGFR is a key molecule orchestrating cell growth, survival, cell cycle progression, and migration and is over-expressed or dysregulated in many cancers^46–49^. EGFR exerts these functions through multiple signaling cascades. After ligand binding, tyrosine residues at the intracellular domain of EGFR are auto-phosphorylated, thus creating docking sites for adapter proteins, such as Grb2 or GAB1 (4). For example, after binding to EGFR, Grb2 recruits SoS1 (a Ras guanine nucleotide exchange factor) cell membrane localization, thereby activating the Ras/Raf/ERK signaling cascade^50, 51^. AP-1 is a downstream convergence point of different signaling cascades and is required for EGF-induced cell transformation and tumor development. In addition, EGFR has been reported to regulate invasion or migration by regulating MMP expression, in a manner dependent on AP-1 activity^52–54^. MMPs have a critical role in ECM remodeling, embryonic development and wound healing in physiological conditions and facilitate tumor cell migration and metastasis through ECM degradation, modulation of cell adhesion and promotion of angiogenesis. We found that AP-1 activity was decreased in both TRAF6-DN and TRAF6-knockdown cells (Fig. 4c), and knockdown of TRAF6 significantly attenuated MMP-2,9 expression upon EGF stimulation (Fig. 4d).

The cSCC is a common skin cancer, and cSCC incidence has increased over the past few decades^55^. Genetic alterations and signaling pathway dysregulation have been found to be associated with cSCC initiation and progression, including upregulation of EGFR, Stat3, and CD147^56–58^. Our results demonstrated that knockdown of TRAF6 decreases A431 cell proliferation and migration (Fig. 2a, d, e), induces cell cycle G2/M arrest (Fig. 2b) and increases p21 transcriptional expression (Fig. 2c). Previous studies have shown that blocking the EGFR/Stat3 axis leads to upregulation of p21 expression^59^, and p21 binds to cyclin-dependent kinases (CDKs) and inhibits CDKs activity, thus leading to growth arrest at G2 phase of the cell cycle^60, 61^. CD147/BSG, a transmembrane glycoprotein belonging to the immunoglobulin superfamily, has various physiological functions, such as fertilization, spermatogenesis, T-cell development, formation of the blood brain barrier and retinal development^62^. CD147 expression had been found to be elevated in different tumors, such as liver, pancreatic, lung, and colon cancers^63–65^. Moreover, CD147 is overexpressed in both head and neck cancer and oral squamous cell carcinomas and is associated with head and neck cancer metastasis and patient survival^66–69^. Interestingly, blocking CD147 expression with anti-CD147 mAb or si-CD147 inhibits EGFR expression and leads to cSCC growth suppression in vitro and in vivo^70^. Targeting both EGFR and CD147 efficiently decreases HNSCC cell proliferation and migration^71^. Our results showed that CD147 directly associates with EGFR, as well as with TRAF6 in HaCaT and A431 cells (Fig. 5a). Notably, knockdown of TRAF6 impairs the interaction between CD147 and EGFR (Fig. 5b) and attenuates EGF-induced EGFR activation (Fig. 5c), in agreement with previous results. In addition, silencing TRAF6 and CD147 expression accelerated EGFR degradation (Fig. 5d) and induced the formation of EGFR-containing endocytic vesicles (Fig. 5e). EGF-induced activation of EGFR and subsequent tyrosine phosphorylation leads to endocytosis of the EGF/EGFR complex into late endosomes, which is followed by lysosomal fusion and degradation of the receptor, thus decreasing EGFR expression on the cell membrane and ultimately downregulating EGFR signaling^72, 73^.

Overall, our study revealed that in addition to the role of TRAF6 in the TLR/IL-1β pathway, TRAF6 is required for Ras (G12V)- and EGF-induced cellular transformation through EGFR. Silencing TRAF6 expression significantly downregulates AP-1 activity, as well as MMP-2,9 expression after EGF stimulation. Furthermore, we confirmed that TRAF6 promotes a malignant cSCC phenotype, thus affecting cell growth and migration. In addition, CD147 associates with EGFR and TRAF6, and knockdown of TRAF6 disrupts the CD147-EGFR complex. Therefore, TRAF6 might be a novel molecular target for cSCC prevention or therapy.

## Materials and methods

### Reagents and antibodies

The cell culture reagents, such as medium (Dulbecco’s modified Eagle’s medium, DMEM) and fetal bovine serum (FBS) were obtained from Life Technologies, Inc. (Rockville, MD). The TRAF6 antibody (Santa Cruz, CA, USA) was used by 1:500, anti-GAPDH (Proteintech, USA) was prepared at 1:3000; the antibody of c-Myc, CD147 (Santa Cruz, CA, USA) had been prepared by 1:500 dilution; The Flag antibody (Sigma, Germany) was diluted at 1:5000 and the no-EGFR, p-EGFR, no-STAT3, p-STAT3 (Cell Signaling Technology, Danvers, MA) were used at 1:1000 dilution. The EGFR Signaling Antibody Array Kit (#12622) was purchased from Cell Signaling Technology (Danvers) Co., Ltd.

### Cell culture and transfections

The NIH3T3, Immortalized human keratinocyte cells (HaCaT) and human epidermoid carcinoma cells (A431) were purchased from the American Type Culture Collection (ATCC) and maintained in DMEM medium (10% FBS, 1% penicillin–streptomycin). The cytogenetically testing and authentication was performed by STR-PCR. For transfection experiments, cells were co-transfected with different plasmids using TurboFect (Thermo Scientific, MA, USA) following the manufacturer’s protocol.

### Lentivirus infection

The stable knockdown TRAF6 expression cells in HaCaT or A431 were generated as described previous^74^. Briefly, pLKO1-TRAF6 plasmids together with pspAX2 and pMD2G were transfected into 293T cells, after 48 and 72 h transfection, the supernatant containing lentiviruses was collected, and then, along with 10 μg/ml polybrene, was perfomred to infect A431 and HaCaT cells. After infection 16 h, the infected cells were continued to culture in medium with 1.0 μg/ml puromycin until the control cells (uninfected) died (usually 2–3 d).

### Immunoprecipitation

The extracts from cells were lysed with NP40 buffer containing protease inhibitor cocktail. For immunoprecipitation, extracts were pretreatment with 20 μl of agarose A/G-Sepharose beads (Beyotime Institute of Biotechnology) and incubated for 1 h at 4 °C with gentle shaking. Supernatants were collected and removed to a new tube, and then, 1.5 μg of antibodies and 40 μl of agarose A/G-Sepharose beads were added to the supernatants, and the mixture was rotated overnight at 4 °C. The beads were then washed three times in NP40 buffer, and the proteins analyzed by western blotting.

### Immunoblotting and antibody array

The cells were collected and lysed by modified RIPA buffer containing 50 mM Tris-Cl pH 8.0, 150 mM NaCl, 0.5% NP-40, and protease-inhibitor cocktail. The protein of concentration had been tested with a BCA Kit, and appropriate amounts of protein were prepared for SDS-PAGE and then transferred to a PVDF membrane (Millipore). The membranes were blocked for 1 h with 5% non-fat dry milk, and then incubated with primary antibodies for overnight at 4 °C. The membranes washed with phosphate-buffered saline (PBS) buffer with 0.1% Tween 20 (PBS-T), reacted with horseradish peroxidase-conjugated secondary antibodies for 1 h, and visualized using an enhanced chemiluminescence substrate. The HaCaT cells were grown to 90% confluence and treated with serum-free medium for overnight. After treated with EGF at 100 ng/ml, cell extracts were prepared and analyzed by PathScan EGFR Signaling Antibody Array Kit (#12622). The antibody array was performed according to the kit protocols recommended by the manufacturer and detected with an imaging system (Bio-Rad, USA).

### H-Ras(G12V) mediates cell transformation

The protocol of H-Ras(G12V) mediated cellular transformation followed previous described^75^. In brief, the H-RasG12V (100 ng) and TRAF6 (2.0 μg) were transiently transfected into NIH3T3 cells at six wells. After transfection 36 h, cells were splited into 10 cm dishes and cultured in DMEM(5% calf serum) for 2 week. The medium had been changed every 3 d. The Foci were fixed and stained with 0.5% crystal violet. Cell colony was calculated with a microscope (×40) and each assay was performed in triplicate.

### Luciferase reporter gene assays

Cells were transfected with *AP-1-Luc* and *SV-40-Renilla-Luc* (Promega, Madison, WI). After 20 h transfection, cells were continued to culture with serum-free medium for 16 h and then treated with EGF (100 ng/ml) for indicated time points. Cell lysates were analyzed by firefly and *Renilla luciferase* activities with a dual-luciferase assay kit (Promega) following protocol.

### Reverse transcription-real time PCR

The RNA was prepared from TRAF6 knocking-down HaCaT cells by the Trizol reagent. A total of 3 µg RNA was used as a template for the reverse transcription reaction (Invitrogen). The *p21* primers used were as follows: (forward) 5′-ACTACCACTCACCCGCAGAC-3′ and (reverse) 5′-CCAGGTCCGTGCAGAAGT-3′.

### Anchorage-independent cell growth

The EGF-induced cell transformation assay was performed as mentioned previous^76^. Cells mixed with Basal Medium Eagle (BME) medium containing 10% FBS and 0.33% agar, in presence of EGF or not, and then, seeded into 6-well plates (8 × 10^3^/ml/well) with coated with 3 ml of solidified BME medium(10% FBS and 0.5% agar). Colonies were scored with a microscope and ImageJ software.

### MTS for cell growth assay

The 2 × 10^3^/well cells were cultured in 96-well plates in 100 µl DMEM; living cells were counted from 0 to 96 h by analyzed with 20 μl of sterile MTS dye each day. Colorimetric analysis was performed at 490 nm was measured using a microplate reader. Each cell line was established in five replicates, and each analysis was repeated three times.

### In vivo tumor growth

Xenograft tumor models were established as mentioned previously^74, 77^, and the animal protocol was approved by the Ethics Committee of Xiangya Hospital (Central South University, China). The A431 cells with knockdown of TRAF6 expression by *sh-Mock* or *sh-TRAF6#1&#2* were collected and washed by PBS, and then resuspended by serum-free medium, the 4 or 6-weeks-old male nude mice (Shanghai SLAC Laboratory Animal Co. Ltd., Shanghai, China) had been subcutaneously injected with tumor cells (5 × 10^6^/0.15 ml) on the right flanks. Tumor diameters and weights were recorded twice a week, and tumor volumes (mm^3^) were calculated by length × width × height × 0.5326. Tumor growth curves were drawn for each group (mean ± S.D.).

### Cell transwell assay

To evaluate the ability of migration in HaCaT and A431 cell lines, an 8-mm-pore size chamber (BD Biosciences, Franklin Lakes, NJ, USA) was used. Briefly, a number of 5 × 10^4^ cells in serum-free medium were put into the upper chamber, and then 500 µl of DMEM plus 20% FBS was added into the lower chambers. After a 16 h of incubation at the temperature of 37 °C, cells in the chamber were fixed with formaldehyde for 15 min and stained with 0.1% crystal violet. Cells passed through the transwell membrane were finally counted with a microscope (×40). Five fields were selected randomly at least. Each assay was performed in triplicate.

### Wound-healing assay

Cells were cultured with complete medium in a 6-well plate at a density of 1 × 10^5^ cells/well, and a straight line were scratched on the cell monolayer was by a 200-μl pipette tip. Then cells were washed by PBS three times to remove debris. Finally, cells were cultured in complete medium and photographed at 24 h and 48 h.

### Cell cycle analysis

Collecting cells first, and washing with PBS, then fixing in ice-cold 70% ethanol in PBS and centrifuged at 4 °C overnight. Next, cells were centrifuged, resuspended with cold PBS and incubated in RNase at 37 °C for 30 min. Then cells were added with propidium iodide and incubated for 30 min at 37 °C. The cell cycle distribution was analyzed by flow cytometry (FCM).

### Immunohistochemistry

Tumor samples from nude mice were cut and mounted on glass slides. Antigen retrieval was operated at 95 °C for 5 min in the microwave oven and cooling to room temperature, deparaffinized sections were treated with 3% hydrogen peroxide for 10 min. The slices were then washed in PBS for three times, 5 min each, and blocked in goat serum for 1 h. This was followed by the addition of anti-Ki-67 or EGFR antibody (1:200, Santa Cruz Biotechnology) in a humidified chamber overnight at 4 °C. After being washed by PBS, the slices were applied with a biotin-conjugated secondary antibody for 20 min and then peroxidase-conjugated streptavidin for an additional 30 min. After that, 3,3′-diaminobenzidine tetrahydrochloride was used to visualize the reaction and then the slices were counterstained with hematoxylin. Immunohistochemistry was performed at least three times.

### Immunofluorescence analysis

Cells seeded in 18-mm cover glasses were washed with ice-cold 1× PBS. After EGF stimulation, 4% paraformaldehyde fixed cells for 15 min at room temperature. Cells were washed by 1× PBS for three times and then permeabilized with 0.5% Triton X-100 for 15 min at room temperature. Cells were blocked by 1% BSA for 15 min and incubated with anti-EGFR (Cell Signaling Technology, #5616) in 1% BSA overnight at 4 °C. Afterwards, the cover glasses were washed by 1× PBS for three times. Cells were counterstained with DAPI (0.5 μg/ml) for 30 min at room temperature. After being washed, the cover glass was mounted through fluorescent mounting medium. Thereafter, fluorescence microscopy images of cells were captured using a Leica SP8 Confocal laser scanning microscope.

### Statistical analysis methods

The statistical results were represented with mean ± S.D. and analyzed by Student’s *t*-test or one-way ANOVA to examine the statistical differences. The *p*-value <0.05 was believed to be statistically significant.

## Electronic supplementary material

supplementary figure legend

sFigure 1
