# Supplementary material for: TRAF6 regulates EGF-induced cell transformation and cSCC malignant phenotype through CD147/EGFR
Source: Oncogenesis. 2018 Feb 20;7(2):17. doi: 10.1038/s41389-018-0030-1 (PMC5833715; doi:10.1038/s41389-018-0030-1)
Supplement: Supplementary file 1 — supplementary figure legend [file 41389_2018_30_MOESM1_ESM.doc]

**TRAF6 regulates EGF-induced cell transformation and cSCC malignant phenotype through CD147/EGFR**

Xu Zhang^1,2^, Lisha Wu^1,2^, Ta Xiao^1,2^, Ling Tang^1,2^, Xuekun Jia^1,2^, Yeye Guo^1,2^, JiangLin Zhang^1,2^, Jie Li^1,2^, Yijing he^1,2^, Juan Su^1,2^, Shuang Zhao^1,2^, Juan Tao^3^, Jianda Zhou^4^, Xiang Chen^1,2#^ and Cong Peng^1,2#^

^1^ The Department of Dermatology, Xiangya Hospital, Central South University, Changsha, Hunan, China

^2^ Hunan Key Laboratory of Skin Cancer and Psoriasis, Xiangya Hospital, Central South University, Changsha, Hunan, China

^3^ Department of Dermatology, Affiliated Union Hospital, Tongji Medical College, Huazhong University of Science and Technology, Wuhan, China.

^4^ Department of Plastic Surgery of Third Xiangya Hospital, Central South University, Changsha, China.

#Address correspondence to: Cong Peng, M.D., Ph.D., The department of Dermatology, Xiangya Hospital, Central South University, Xiangya Road #87, Changsha, Hunan, China, 410008. Tel: +86-731-84327377; FAX: +86-731-84328478; Email:pengcongxy@csu.edu.cn

# Address correspondence to: Xiang Chen, M.D., Dr. P.H., The department of Dermatology, Xiangya Hospital, Central South University, Xiangya Road #87, Changsha, Hunan, China, 410008. Tel: +86-731-84327377; FAX: +86-731-84328478;

Email: chenxiangck@126.com

**Running Title: TRAF6 regulates EGFR expression**

**Supplementary Figure Legend**

**Figure 1. The activation of TRAF6 E3 ligase is required for cell proliferation and migration. (a)** TRAF6-wt or TRAF6-DN were transfected into TRAF6 knock down HaCaT cells as described in *Materials and Methods*. The protein levels of transfected TRAF6–wt and TRAF6-DN were tested by western blotting with the indicated antibodies. **(b)** The TRAF6-wt and TRAF6-DN cells were seeded into 96-well plates, and proliferation was assessed by using a CellTiter96 Aqueous One Solution detection kit. Data from multiple experiments are expressed as the means ± S.D. Significant differences were evaluated using one-way ANOVA, and the asterisk (*) indicates a significant difference (p < 0.05). **(c)** The effect of TRAF6-DN on cell migration. Cells that migrated across the membrane were stained with crystal violet and imaged at 100x magnification (left panel). The data represent the means (n=5) ±SD of each group. The asterisk (*) indicates a significant difference between cells expressing *mock* or *TRAF6-wt and TRAF6-DN* (p < 0.05, one-way ANOVA)(right panel).
