# Supplementary figures and images for: TRAF6 regulates EGF-induced cell transformation and cSCC malignant phenotype through CD147/EGFR
Source: Oncogenesis. 2018 Feb 20;7(2):17. doi: 10.1038/s41389-018-0030-1 (PMC5833715; doi:10.1038/s41389-018-0030-1)

# Supplementary Figure 1 a-d

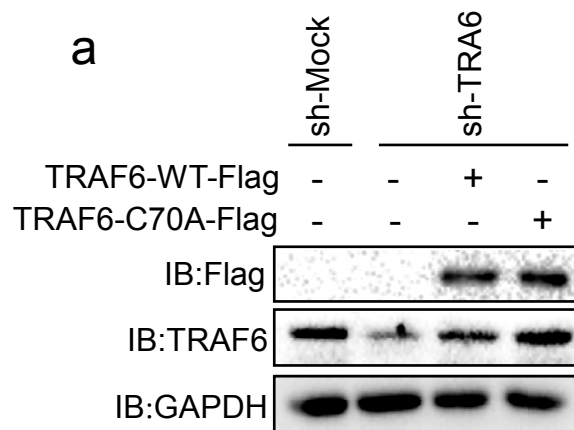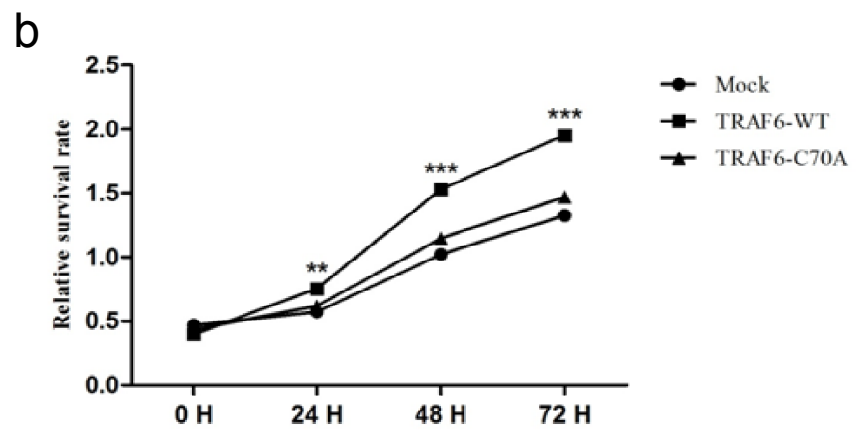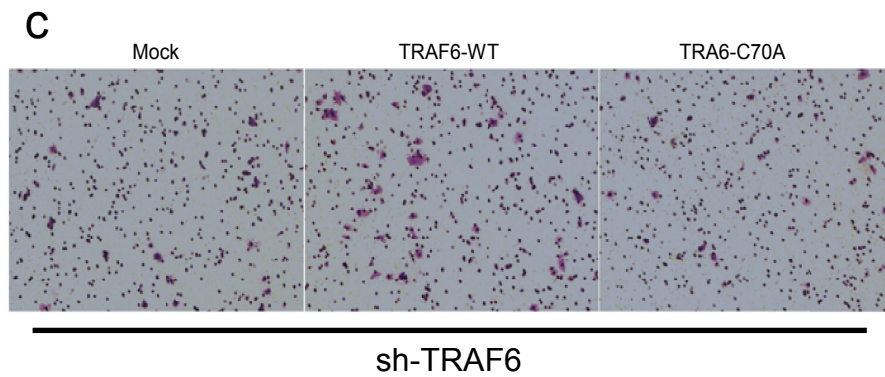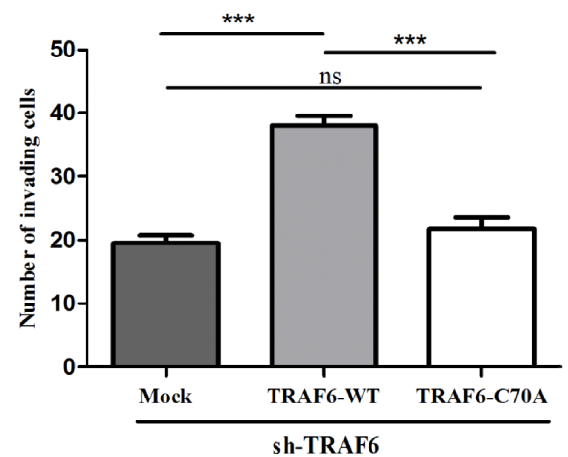

Supplement: Supplementary file 2 — sFigure 1 [file 41389_2018_30_MOESM2_ESM.pdf]
